# Using Deep Learning Radiomics to Distinguish Cognitively Normal Adults at Risk of Alzheimer’s Disease From Normal Control: An Exploratory Study Based on Structural MRI

**DOI:** 10.3389/fmed.2022.894726

**Published:** 2022-04-21

**Authors:** Jiehui Jiang, Jieming Zhang, Zhuoyuan Li, Lanlan Li, Bingcang Huang, Alzheimer’s Disease Neuroimaging Initiative

**Affiliations:** ^1^Department of Radiology, Gongli Hospital, School of Medicine, Shanghai University, Shanghai, China; ^2^School of Life Sciences, Institute of Biomedical Engineering, Shanghai University, Shanghai, China; ^3^School of Communication and Information Engineering, Shanghai University, Shanghai, China

**Keywords:** deep learning radiomic, Alzheimer’s disease, magnetic resonance imaging, support vector machine, artificial intelligence

## Abstract

**Objectives:**

We proposed a novel deep learning radiomics (DLR) method to distinguish cognitively normal adults at risk of Alzheimer’s disease (AD) from normal control based on T1-weighted structural MRI images.

**Methods:**

In this study, we selected MRI data from the Alzheimer’s Disease Neuroimaging Initiative Database (ADNI), which included 417 cognitively normal adults. These subjects were divided into 181 individuals at risk of Alzheimer’s disease (preAD group) and 236 normal control individuals (NC group) according to standard uptake ratio >1.18 calculated by amyloid Positron Emission Tomography (PET). We further divided the preaAD group into APOE+ and APOE**−** subgroups according to whether APOE ε4 was positive or not. All data sets were divided into one training/validation group and one independent test group. The proposed DLR method included three steps: (1) the pre-training of basic deep learning (DL) models, (2) the extraction, selection and fusion of DLR features, and (3) classification. The support vector machine (SVM) was used as the classifier. In the comparative experiments, we compared our proposed DLR method with three existing models: hippocampal model, clinical model, and traditional radiomics model. Ten-fold cross-validation was performed with 100 time repetitions.

**Results:**

The DLR method achieved the best classification performance between preAD and NC than other models with an accuracy of 89.85% ± 1.12%. In comparison, the accuracies of the other three models were 72.44% ± 1.37%, 82.00% ± 4.09% and 79.65% ± 2.21%. In addition, the DLR model also showed the best classification performance (85.45% ± 9.04% and 92.80% ± 2.61%) in the subgroup experiment.

**Conclusion:**

The results showed that the DLR method provided a potentially clinical value to distinguish preAD from NC.

## Introduction

Alzheimer’s disease (AD) is a neurodegenerative disease characterized by progressive cognitive decline (1). Due to the irreversibility of AD, it is critical to identify AD patients at an ultra-early stage. According to the latest A-T-N diagnosis criteria (2–4), individuals who showed obvious brain amyloid beta (Aβ+) deposition have entered the Alzheimer’s continuum and represented a high-risk of AD. This population could be defined as the preclinical AD group (PreAD) (5, 6).

So far, structural resonance imaging (MRI) have been widely used in the diagnosis of AD (7–11). For instance, previous studies have shown that patients with mild cognitive impairment(MCI) had increased hippocampal atrophy compared to normal control (NC) subjects (12). The atrophy of hippocampal and entorhinal cortex could also be used as an index to predict the conversion from MCI to AD (13).

Currently, artificial intelligence (AI) techniques based on MRI have frequently been used in the early diagnosis of AD. One typical AI application is radiomics. For example, Zhao et al. investigated hippocampal texture radiomics features as effective MRI biomarkers for AD and achieved an accuracy of 87.4% to distinguish AD and normal controls (NC) (14). Zhou and Shu et al. utilized MRI radiomics features to predict development of MCI to AD and achieved the accuracy of 78.4 and 80.7%, respectively (15, 16). Notably, Li et al. conducted an exploratory study to diagnosis preAD from NC based on radiomics multi-parameter MRI and obtained an average accuracy of 83.7% [T. (17)]. Although the feasibility of traditional radiomics methods has been proven, these methods could not be widely applied because of obvious shortcomings, such as manual extraction of regions of interest (ROIs) and hand-coding, which usually require complex manual operations. Therefore, an alternative method is required.

The deep learning radiomics (DLR) method may be the alternative (18, 19). This technique was able to mine the high dimension features of medical images automatically, and effectively address the shortage of hand-coding by radiomics. Recently, DLR has been used in brain tumor-related research and AD diagnosis (20, 21). For example, previous studies achieved good predictive performance of preoperative meningioma with an accuracy of 92.6% (22). Wang et al. extracted MRI-based DLR features to predict the prognosis of high-grade glioma (23). For AD diagonosis, early DLR-based methods always focused on pre-determined regions of interest prior to deep training, which may hamper diagnostic performance. For example, Khvostikov et al. and Li et al. trained DLR models based on pre-extracted hippocampal regions of MRI and other multimodal neuroimaging data (24, 25). Apart from the above, Basaia et al. used a single cross-sectional MRI scan and deep neural networks to automatically classify AD and MCI, with high accuracies of 98.2% between AD and NC, and of 74.9% from MCI to AD progression (26). Lee et al. also used DLR method for AD classification and achieved the accuracies of 95.35% and 98.74% on different datasets (27). However, there is no existing DLR model for preAD detection.

Therefore, in this study we hypothesized that the DLR method was useful in the diagnosis of PreAD. Considering the hippocampus volume has not been shrunk in AD early stage, we used MRI images of the whole brain for DLR classification. In addition, we also hypothesized that the DLR technique could achieve high classification accuracy in detecting subgroups of preAD from NC, such as APOE ε4+ individuals.

## Materials and Methods

**FIGURE 1:**
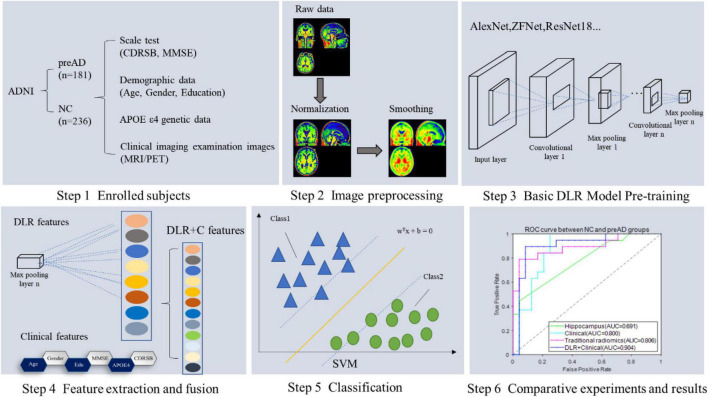
The framework of this study.

Figure 1 showed the overall framework of this study, which consisted of six steps: (1) enrolled subjects; (2) imaging preprocessing, including segmentation, normalization and smoothing; (3) basic deep learning (DL) model pre-training, in this step several DL models were pre-trained in order to get the best one for DLR feature extraction; (4) feature extraction and fusion; (5) classification; (6) comparative experiments.

### Subjects

The data used in this study were obtained from the Alzheimer’s Disease Neuroimaging Initiative (ADNI) database.^1^ ADNI is a longitudinal, multicenter study to develop clinical, imaging, genetic and biochemical biomarkers for early detection and tracking of AD. The latest information is available at http://adni.loni.usc.edu/about/.

In this study, we collected 236 NC and 181 preAD data. Demographic data included age, sex, gender, education, neuropsychological assessment tests [Dementia Rating Scale (CDRSB) and Mini-Mental State Examination (MMSE)], Apolipoprotein E (APOE) ε4 and imaging information. T1-MRI and amyloid positron emission tomography (PET) images were selected for all subjects. The preAD group was defined as who standard uptake value ratio (SUVR) value of amyloid PET was >1.18 in whole cerebral cortex (17, 28). Whole cerebellum was used for reference when deriving SUVR. In addition, to validate our proposed DLR model, we enrolled 12 preAD individuals who converted into the MCI state. We selected MRI images in both baseline and MCI stages.

All subjects were divided into two groups, one training/validation group and one independent test group. The training/validation group was from ADNI 1, ADNI 2 and ADNI 3, including 212 NC and 162 preAD subjects. The test group was from ADNI Go, including 24 NC subjects and 19 preAD subjects.

### Images Acquisition and Preprocessing

The image acquisition process was described in the ADNI website at http://adni.loni.usc.edu/about/. All MRI data have been evaluated by quality control (QC) at the Mayo Clinic Aging and Dementia Imaging Research Laboratory. The SUVR values of Amyloid PET were downloaded from the ADNI website directly.

The preprocessing of MRI images was performed by statistical parametric mapping (SPM12) software^2^ on MATLAB 2016b platform.^3^ First, MRI images were segmented into probabilistic gray matter (GM), white matter (WM), and cerebrospinal fluid (CSF); Then, each GM image was normalized into the Montreal Neurological Institute (MNI) space by diffeomorphic anatomical registration *via* exponentiated lie algebra, and smoothed using an 8-mm Gaussian-smoothing kernel. As a result, each image has a spatial resolution of 91 × 109 × 91 with a voxel size of 2 mm × 2 mm × 2 mm; Finally, in order to adapt and speed up the training of the deep learning model, 3D images were sliced from the axial direction into 91 single-channel images with the size of 91 × 109 to tile 2D images, and then resized into 224 × 224 for normalizing. Each 3D MRI image was tiled into a group of 2D images and resized into 224 × 224 pixels for subsequent DL model training.

### The Proposed Deep Learning Radiomics Method

**FIGURE 2:**
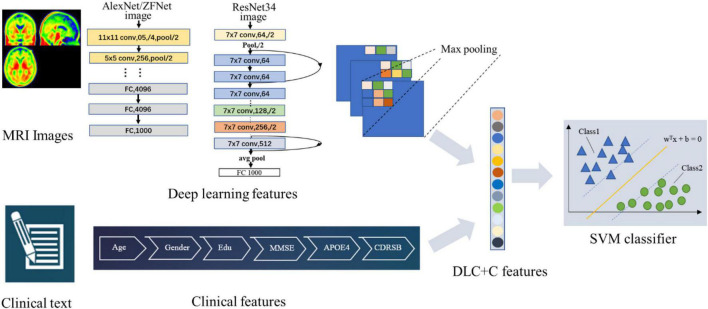
The flow chart of the proposed DLR model.

Figure 2 illustrates our proposed DLR method. The method consisted of three parts: (1) Basic DL model pre-training. We used six Convolutional Neural Networks (CNN) networks as candidate DL models and pre-trained them, respectively. After training, we selected one as the final DL model to obtain the DLR features according to the classification results. (2) Feature fusion. To obtain DLR features, we obtained DLR feature maps from the last convolutional layer of the final selected DL model, and extracted the maximum value of each feature map through global max pooling. These extracted features were defined as DLR features and combined with clinical features (sex, education, etc.) as input data for classification. (3) Classification. Based on the above features, the support vector machine (SVM) was used as the classifier to distinguish preAD from NC.

#### Training for Candidate Deep Learning Models

Six CNN models, including AlexNet, ZFNet, ResNet18, ResNet34, InceptionV3, and Xception, were applied in the training step to define the best training model.

• AlexNet: it is the first CNN network architecture that uses ReLU as the activation function, and uses interleaving pooling technology in CNN (29).
• ZF-Net: it is fine-tuned on the basis of AlexNet. It uses deconvolution to visually analyze the intermediate feature map of CNN and improves model performance by analyzing feature behavior (30).
• Inceptionv3: it improves the CNN model by using convolution decomposition and regularization (31).
• Xception: it improves Inception V3 by using depth wise separable convolution to replace the Inception module (32, 33).
• ResNet: it introduces new network features based on the previous traditional CNN network (34). Several ResNet subtypes were proposed according to different numbers of hidden layers, such as ResNet18, ResNet34, ResNet101, and so on.

**FIGURE 3:**
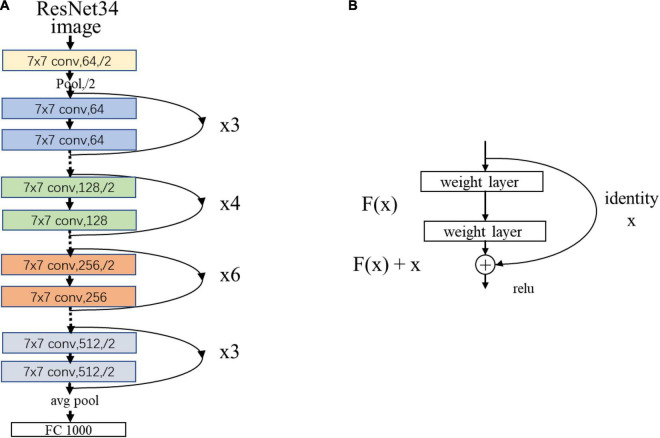
**(A)** The network structure of the ResNet34 model. “7 × 7” represents the size of the convolution kernel, “conv” represents convolution, “avg pool” represents average pooling, and “fc” represents fully connected layer. “64” means the number of channels, and “/2” means stride of 2. **(B)** Residual learning: a building block. x represents direct identity mapping, F(x) represents residual mapping, and F(x)+x is output.

As an example, Figure 3 showed the network structure of the ResNet34 model.

During the raining step, the selected six models were trained in the training/validation group and tested in the test group. Guided by the test results, we optimized the DL model by tuning hyper parameters.

#### Classifier

We combined DLR features and clinical information (gender, education, age, etc.) as input data for classification. SVM was used as the classifier. As a classic supervised learning method, SVM has been widely used in statistical classification and regression analysis due to its ability to map vectors to a higher dimensional space that creates a maximum margin hyperplane to achieve high classification performance (35). In this study, we used the linear kernel function in SVM to detect classification reliability and generalization ability.

### Comparative Experiments

To demonstrate the superiority of our proposed DLR method, we compared our model and three existing models in comparative experiments, including: (1) Clinical model: clinical characteristics included demographic data, neuropsychological cognitive assessment results, and APOE ε4 genotyping characteristics of all subjects. (2) Hippocampal model: the hippocampal volumes were used as inputs for the classification; (3) Traditional radiomics model: traditional radiomics features of were extracted for the classification. In this experiment, we extracted features by using the radiomics tool developed by Vallieres et al.^4^ We used brain DMN regions as ROIs and performed texture analysis on each input ROI using the “Texture Toolbox” in the Radiomics Toolbox. Feature extraction steps included wavelet bandpass filtering, isotropic resampling, Lloyd–Max quantization and feature calculation. The detailed extraction process of the radiomics features were described in the previous studies (36, 37).

Three comparative experiments were employed in this study: (1) NC vs. preAD; (2) NC vs. preAD APOE+; and (3) NC vs. preAD APOE−. Ten-fold cross-validation was performed with 100 time repetitions. We calculated accuracy, sensitivity, and specificity to evaluate the classification results. The mathematical expressions of the three indicators were as follows:


$$\mathrm{Accuracy}=\frac{\mathrm{TP}}{\mathrm{TP}+\mathrm{FP}}$$

$$\mathrm{Sensitivity}=\frac{\mathrm{TP}}{\mathrm{TP}+\mathrm{FN}}$$

$$\mathrm{Specificity}=\frac{\mathrm{TN}}{\mathrm{FP}+\mathrm{TN}}$$

### Longitudinal Study

The 12 individuals with longitudinal data were used to validate the proposed DLR model. Firstly, we calculated the probability value of SVM classifier, and defined it as the decision score; then we compared the decision scores in both baseline and MCI states in 12 individuals.

### Statistical Analysis

In this study, we used two-sample *t*-tests or chi-square tests to compare demographic and clinical characteristics between the NC and preAD groups and between the APOE+ and APOE**−** subgroups. All statistical analyses were performed using SPSS version 22.0 software (SPSS Inc., Chicago, IL, United States) and performed in Matlab2019b (Mathworks Inc., Sherborn, MA, United States). A *p*-value < 0.05 was considered to be significantly different.

## Results

### Demographic Information

The results of demographic data were shown in Table 1. There was a significant difference in age and years of education between the preAD group and the NC group in the training/validation group (*p* < 0.001), and there was a difference in CDRSB and MMSE (CDRSB: *p* = 0.006, MMSE: *p* = 0.003), while no difference in gender between the two groups. There was no significant difference in gender, education level, CDRSB and MMSE between the preAD group and the NC group in the test group, whereas there was a difference in age (*p* = 0.03).

**TABLE 1:** Demographic information for subjects.

	Training/validation groups				Test groups				Longitudinal data
	preAD	APOE+	APOE−	NC	preAD	APOE+	APOE−	Baseline	MCI
N	162	70	92	212	19	9	10	16	16
Gender(M/F)	68/94	36/34	32/60	103/109	5/14	3/6	2/8	9/7	9/7
Age(years)	76.3 ± 5.4	75.3 ± 6.3	76.9 ± 4.5	71.8 ± 5.7*^b^*	75.3 ± 5.1	74.7 ± 6.9	75.9 ± 3.4	71.5 ± 5.8*^b^*	80.8 ± 5.4
EDU	15.4 ± 3.0	14.9 ± 3.5	15.8 ± 2.5	16.7 ± 2.5*^b^*	15.4 ± 2.1	16.0 ± 2.4	14.8 ± 1.7	16.13 ± 2.4	16.13 ± 2.4
MMSE	28.7 ± 1.6	28.5 ± 1.6	28.8 ± 1.6	29.1 ± 1.3*^b^*	28.7 ± 1.3	28.8 ± 1.1	28.6 ± 1.6	29.2 ± 0.9	27.43 ± 2.0
CDRSB	0.3 ± 0.7	0.3 ± 0.8	0.3 ± 0.7	0.2 ± 0.4*^b^*	0.3 ± 0.9	0.5 ± 1.2	0.1 ± 0.2	0.1 ± 0.2	1.63 ± 0.9
APOE ε4 positive rate	70/162	N/A	N/A	34/212	9/19	N/A	N/A	3/13	3/13

*All data except APOEε4 positive rate were presented as mean ± standard deviation. EDU, education; MMSE, Mini-mental State Examination; CDRSB, clinical dementia rating sum of boxes.*

*^a^Age, Education, MMSE and CDRSB performed a two-sample t-test between NC and preAD groups; Gender performed a Chi-square test between NC and preAD groups.*

*^b^Means that there was a significant difference (p < 0.05) between the preAD group and the NC group in the training/validation group and test group with two-sample t-tests.*

### Pre-training for Candidate Deep Learning Models

Table 2 summarized the classification performance of six candidate DL models, including classification accuracy, sensitivity, and specificity. By comparing the results of the two groups, ResNet34 was selected to be the best model. Therefore, we chose the pre-training ResNet34 model and extracted DLR features for the next step.

**TABLE 2:** Classification performance of different DL models in the pre-training step.

Model	Accuracy (%)	Sensitivity (%)	Specificity (%)	AUC
**Training/Validation Groups**				
AlexNet	96.28 ± 3.24	94.86 ± 5.88	97.38 ± 2.46	0.962 ± 0.04
ZF-Net	98.18 ± 1.88	97.55 ± 3.50	98.83 ± 1.98	0.980 ± 0.02
ResNet18	95.68 ± 2.66	94.49 ± 4.93	96.58 ± 3.05	0.962 ± 0.03
ResNet34	**96.29 ± 2.54**	**96.62 ± 2.26**	**96.02 ± 3.58**	0.964 ± 0.02
InceptionV3	97.63 ± 2.43	95.91 ± 4.99	98.95 ± 1.35	0.976 ± 0.01
Xception	97.02 ± 3.84	97.62 ± 3.62	96.54 ± 5.15	0.973 ± 0.03
**Test Groups**				
AlexNet	87.91 ± 3.06	78.95 ± 4.30	95.00 ± 3.83	0.869 ± 0.03
ZF-Net	87.91 ± 2.40	79.47 ± 3.88	94.58 ± 2.01	0.870 ± 0.03
ResNet18	87.67 ± 1.91	84.21 ± 3.50	90.41 ± 2.01	0.872 ± 0.02
ResNet34	**89.53 ± 2.51**	**87.89 ± 2.54**	**90.83 ± 5.12**	0.893 ± 0.03
InceptionV3	84.88 ± 2.26	84.21 ± 3.51	85.42 ± 4.05	0.848 ± 0.03
Xception	88.84 ± 2.14	88.40 ± 3.30	89.17 ± 4.48	0.886 ± 0.04

*The bold values indicate classification results of the optimal model ResNet34 for Base DLR Model Selection.*

### Comparative Experiments

#### Normal Control vs. Preclinical Alzheimer’s Disease Group

Table 3 showed the classification results of the four models between NC and preAD groups. Among the four models, the DLR model showed the best classification performance in the test group, with the accuracy of 89.85% ± 1.12%, sensitivity of 94.74% ± 0.1%, and specificity of 85.98% ± 2.01%. The performance of the hippocampal model, traditional radiomics model, and clinical model were all significantly lower than DLR model, with the accuracies of 72.44% ± 1.37%, 82.00% ± 4.09% and 79.65% ± 2.21%, sensitivities of 42.68% ± 2.93%, 68.59% ± 8.35% and 82.754% ± 4.24%, specificities of 96.09% ± 1.31%, 92.62% ± 4.58% and 77.20% ± 2.61%, respectively.

**TABLE 3:** The classification results of preAD vs. NC.

Model	Accuracy (%)	Sensitivity (%)	Specificity (%)
**Training/Validation Groups**			
Hippocampal model	76.20 ± 6.05	44.72 ± 10.58	99.05 ± 2.27
Traditional radiomics model	77.01 ± 7.77	62.61 ± 10.31	87.73 ± 9.50
Clinical model	85.66 ± 5.24	83.31 ± 9.56	87.70 ± 7.65
DLR model	**99.40 ± 3.23**	**99.00 ± 4.00**	**99.56 ± 1.65**
**Test Groups**			
Hippocampal model	72.44 ± 1.46	42.68 ± 2.93	96.09 ± 1.31
Traditional radiomics model	82.00 ± 4.09	68.59 ± 8.35	92.62 ± 4.58
Clinical model	79.65 ± 2.21	82.75 ± 4.24	77.20 ± 2.61
DLR method	**89.85 ± 1.12**	**94.74 ± 0. 10**	**85.98 ± 2.01**

*Bold values represent the classification performance of our proposed model.*

**FIGURE 4:**
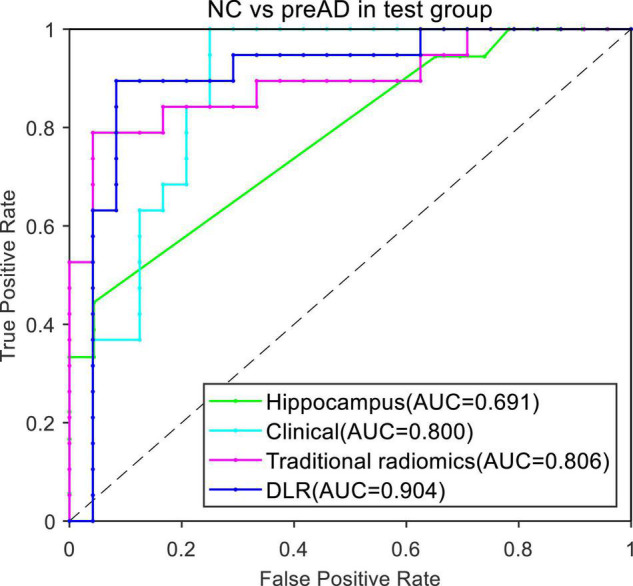
ROC curves of the four models between NC and preAD groups.

Figure 4 presented the ROC curves of the four models. The mean AUCs (± SD) for the hippocampal model, traditional radiomics model, clinical model, and DLR model in were 0.691 ± 0.012, 0.806 ± 0.013, 0.800 ± 0.021 and 0.904 ± 0.014, respectively.

#### Normal Control vs. Preclinical Alzheimer’s Disease Subgroups

Table 4 showed the classification results between NC and preAD APOE+ groups. The accuracy, sensitivity and specificity of the DLR model in the test group were 92.80% ± 2.61%, 88.89% ± 0.01%, and 94.47% ± 3.72%. The performance of the hippocampal model, traditional radiomics model, and clinical model were all significantly lower than our proposed model, with the accuracies of 72.44%.

**TABLE 4:** The classification results of NC vs. preAD APOE+.

Model	Accuracy (%)	Sensitivity (%)	Specificity (%)
**Training/Validation Groups**			
Hippocampal model	76.90 ± 11.62	49.78 ± 26.80	99.37 ± 5.44
Traditional radiomics model	71.11 ± 10.60	54.42 ± 16.69	83.66 ± 9.33
Clinical model	71.11 ± 10.98	50.80 ± 15.66	84.94 ± 12.35
DLR model	**99.94 ± 0.59**	**99.95 ± 0.01**	**99.88 ± 3.72**
**Test Groups**			
Hippocampal model	69.00 ± 6.84	30.71 ± 16.94	96.84 ± 15.91
Traditional radiomics model	78.87 ± 5.00	54.42 ± 16.21	83.66 ± 6.72
Clinical model	71.39 ± 4.65	32.84 ± 13.65	96.17 ± 4.37
DLR model	**92.80 ± 2.61**	**88.89 ± 0.01**	**94.47 ± 3.72**

*Bold values represent the classification performance of our proposed model.*

Table 5 showed the classification results between NC and preAD APOE**−** groups. The accuracy, sensitivity and specificity of the DLR model in the test group were 85.45 ± 9.04%, 90.40% ± 9.47%, and 83.10% ± 11.66%. The performance of the hippocampal model, traditional radiomics model, and clinical model were all significantly lower than our proposed model, with the accuracies of 63.36% ± 7.42%, 83.87% ± 3.04%, and 70.10% ± 3.50%. In Tables 3, 4, the bold values represented the classification performance of the our proposed method.

**TABLE 5:** The classification results of NC vs. preAD APOE−.

Model	Accuracy (%)	Sensitivity (%)	Specificity (%)
**Training/Validation Groups**			
Hippocampal model	76.88 ± 12.86	75.46 ± 23.37	77.83 ± 12.60
Traditional radiomics model	73.50 ± 9.44	73.20 ± 12.45	72.51 ± 12.35
Clinical model	70.28 ± 9.69	60.20 ± 16.61	79.22 ± 12.05
DLR model	**95.74 ± 11.85**	**89.60 ± 10.54**	**98.03 ± 10.76**
**Test Groups**			
Hippocampal model	63.36 ± 7.42	75.82 ± 24.86	50.90 ± 21.02
Traditional radiomics model	83.87 ± 3.04	78.00 ± 11.35	86.67 ± 6.66
Clinical model	70.10 ± 3.50	62.03 ± 7.93	73.95 ± 7.27
DLR model	**85.45 ± 9.04**	**90.40 ± 9.47**	**83.10 ± 11.66**

*Bold values represent the classification performance of our proposed model.*

**FIGURE 5:**
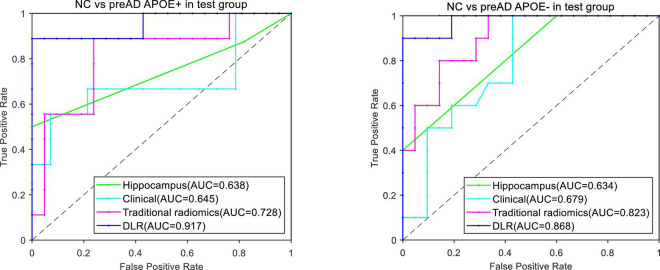
ROC curves of the four models between NC and preAD APOE+ groups **(left)** and between NC and preAD APOE**–** groups **(right)**.

Figure 5 showed the ROC curves of the four models. The mean AUCs (± SD) for the hippocampal model, traditional radiomics model, clinical model and the best DLR model between NC and preAD APOE+ were 0.638 ± 0.061, 0.728 ± 0.024, 0.645 ± 0.041 and 0.917 ± 0.010, and between NC and preAD APOE**−** were 0.634 ± 0.075, 0.823 ± 0.041, 0.679 ± 0.042, and 0.868 ± 0.011, respectively.

### Longitudinal Study

**FIGURE 6:**
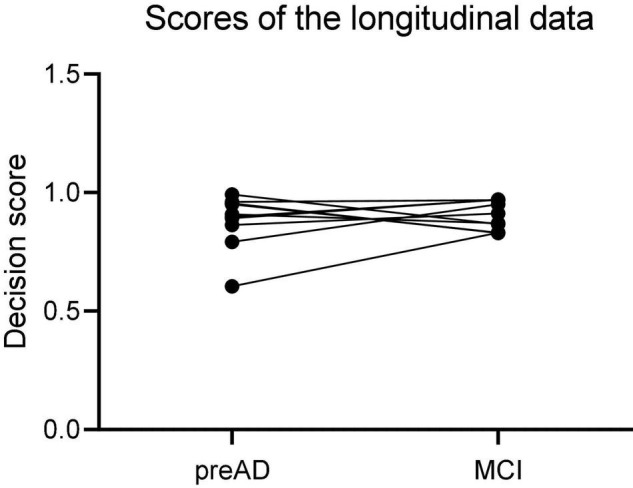
The scores of the longitudinal data in preAD stage and MCI stage.

Figure 6 showed the results of the longitudinal study. The decision scores had a slight upward trend from the PreAD baseline to the MCI stage. The results showed that our model also had a great prediction performance.

## Discussion

Currently, DLR is the hot spot and focus of current imaging development. In view of its superiority in disease diagnosis, DLR methods have been successfully applied in tumor genotype prediction, preoperative analysis, prognosis evaluation, and cancer diagnosis, etc., but DLR research for neurological diseases remained lacking. In this study, we proposed a DLR model to distinguish cognitively normal adults at risk of Alzheimer’s disease from normal control based on T1-weighted structural MRI images. Compared with other traditional models, such as hippocampal model, clinical model or traditional radiomics model, our proposed DLR model achieved best classification results.

In the comparative experiments, the DLR method achieved the highest accuracy in both training/validation group (99.40% ± 3.23%) and separate test group (89.85% ± 1.12%). Therefore, we proved the robustness of the DLR model.

Currently, several studies have investigated the classification between preAD and NC by using machine learning or traditional quantitative methods. For example, Ding et al. distinguished preAD from NC by investigating the coupling relationship between glucose and oxygen metabolism from hybrid PET/MRI, with an AUC of 0.787 (38). Li et al. used a voxel-based SSM/PCA method to analyze fluorodeoxyglucose-PET (FDG-PET) images with AUC of 0.815 (39), Li et al. conducted an exploratory study for identifying preAD based on radiomics analysis of MRI and obtained an average accuracy of 83.7% [T. (17)]. In comparison to previous studies, our DLR model achieved the best classification results. The reason can be explained as following: (1) the DLR method can directly extract high-throughput image features from CNN. Since it does not involve additional feature extraction operations, it will not bring additional errors; (2) the results of traditional methods were usually influenced by individual factors and imaging machine parameters; while the DLR method combined DLR image features and clinical information, which partly solved the problems of individual heterogeneity.

To demonstrate the robustness of the proposed DLR model, we performed experiments in the APOE ε4 subgroup analysis. Notably, cerebral amyloid deposition is also affected by the ApoE ε4 genotype (40). Higher levels of amyloid accumulation were observed in SCD subjects with ApoE ε4 carriers than noncarriers (41, 42). Therefore, we proposed to add ApoE ε4 genotype features to further validate the accuracy of the model. Notably, the DLR model achieved better classification results between NC vs. preAD APOE+ (92.80% ± 2.61%) than the two other experiments (89.85% ± 1.12% and 85.45% ± 9.04%). The high sensitivity (88.89% ± 0.01%) and specificity (94.47% ± 3.72%) results also showed that the DLR model was very powerful in identifying cognitively normal adults at risk of Alzheimer’s disease.

Although the DLR method could distinguish preAD from NC, it still had some limitations. First, more data was still needed to verify the generality and robustness of the proposed method. In this study, subjects were collected only from the ADNI database. Whether our model was powerful for other racial populations need further exploration. Secondly, we only compared six DL models. Although the Resnet34 model achieved good classification performance, it was unknown whether other DL models beyond the six were more suitable. In addition, we used the whole brain MRI image to train the DLR models in this study. However, future studies were required to explore whether DLR models based on the hippocampus or entorhinal cortex instead were more effective. Furthermore, in this study, 2D DLR models were employed. However, whether 3D DLR models could achieve better classification performances need further exploration. Finally, the proposed DLR model was based on T1-MRI images. It may be possible to improve the classification performance of DLR by combining other imaging modals, such as FDG PET, amyloid PET and tau PET images.

## Conclusion

We proposed a DLR method based on T1-MRI images to discriminate preAD and NC. The results demonstrated that our proposed DLR method could improve diagnostic performance. The DLR method had potentials for clinical applications in the future.

## Data Availability Statement

The original contributions presented in the study are included in the article/supplementary material, further inquiries can be directed to the corresponding author.

## Ethics Statement

Written informed consent was obtained from the individual(s) for the publication of any potentially identifiable images or data included in this article.

## Author Contributions

JJ conceived and designed the experiments, analyzed and interpreted the data, and wrote the manuscript. JZ performed the experiments, interpreted the data, and wrote the manuscript. ZL and LL performed the experiments and wrote the manuscript. BH conceived and designed the experiments, provided research funding, analyzed and interpreted the data, and reviewed the manuscript. All authors read and approved the final version of the article for publication.

## Conflict of Interest

The authors declare that this study was conducted without any commercial or financial relationships that could be construed as potential conflicts of interest.

## Publisher’s Note

All claims expressed in this article are solely those of the authors and do not necessarily represent those of their affiliated organizations, or those of the publisher, the editors and the reviewers. Any product that may be evaluated in this article, or claim that may be made by its manufacturer, is not guaranteed or endorsed by the publisher.

## Footnotes

1
https://adni.loni.usc.edu/wp-content/uploads/how_to_apply/ADNI_Acknowledgement_List.pdf

2
https://www.fil.ion.ucl.ac.uk/spm/software/spm12/

3
https://www.mathworks.com/products/matlab.html

4
https://github.com/mvallieres/radiomics
